# Author Correction: The FANCM-BLM-TOP3A-RMI complex suppresses alternative lengthening of telomeres (ALT)

**DOI:** 10.1038/s41467-019-13097-2

**Published:** 2019-11-20

**Authors:** Robert Lu, Julienne J. O’Rourke, Alexander P. Sobinoff, Joshua A. M. Allen, Christopher B. Nelson, Christopher G. Tomlinson, Michael Lee, Roger R. Reddel, Andrew J. Deans, Hilda A. Pickett

**Affiliations:** 1 Telomere Length Regulation Unit, Children’s Medical Research Institute, Faculty of Medicine and Health, University of Sydney, Westmead, 2145 NSW Australia; 2 Genome Stability Unit, St. Vincent’s Institute, 9 Princes St, Fitzroy, 3065 VIC Australia; 3 Department of Medicine (St. Vincent’s), University of Melbourne, Parkville, 3052 VIC Australia; 4 Cancer Research Unit, Children’s Medical Research Institute, Faculty of Medicine and Health, University of Sydney, Westmead, 2145 NSW Australia

**Keywords:** Cancer therapy, Molecular biology

Correction to: *Nature Communications* 10.1038/s41467-019-10180-6, published online 28 May 2019.

The original version of this Article contained an error in the Supplementary Information file. In the figure legend for Supplementary Fig. 6 panel b, “Flag-FANCM immunoprecipitation of the FANCM-BTR complex (TOP3A binding) diminishes with increasing concentrations (1–50 µM) of MM2-WT or MM2-FF>AA mutant peptide in HEK-293 cells expressing Flag-FANCM.” should have read “Flag-FANCM immunoprecipitation of the FANCM-BTR complex (TOP3A binding) diminishes with increasing concentrations (1–50 µM) of MM2-WT, but not with the MM2-FF>AA mutant peptide in HEK-293 cells expressing Flag-FANCM.” This has now been corrected in both the PDF and HTML versions of the Article.

