# Supplementary material for: The FANCM-BLM-TOP3A-RMI complex suppresses alternative lengthening of telomeres (ALT)
Source: Nat Commun. 2019 May 28;10:2252. doi: 10.1038/s41467-019-10180-6 (PMC6538672; doi:10.1038/s41467-019-10180-6)
Supplement: Supplementary file 1 — Supplementary Information [file 41467_2019_10180_MOESM1_ESM.pdf]

Supplementary Information

The FANCM-BLM-TOP3A-RMI complex suppresses alternative lengthening of telomeres (ALT)

Lu et al.

Supplementary Figures

Supplementary Fig. 1

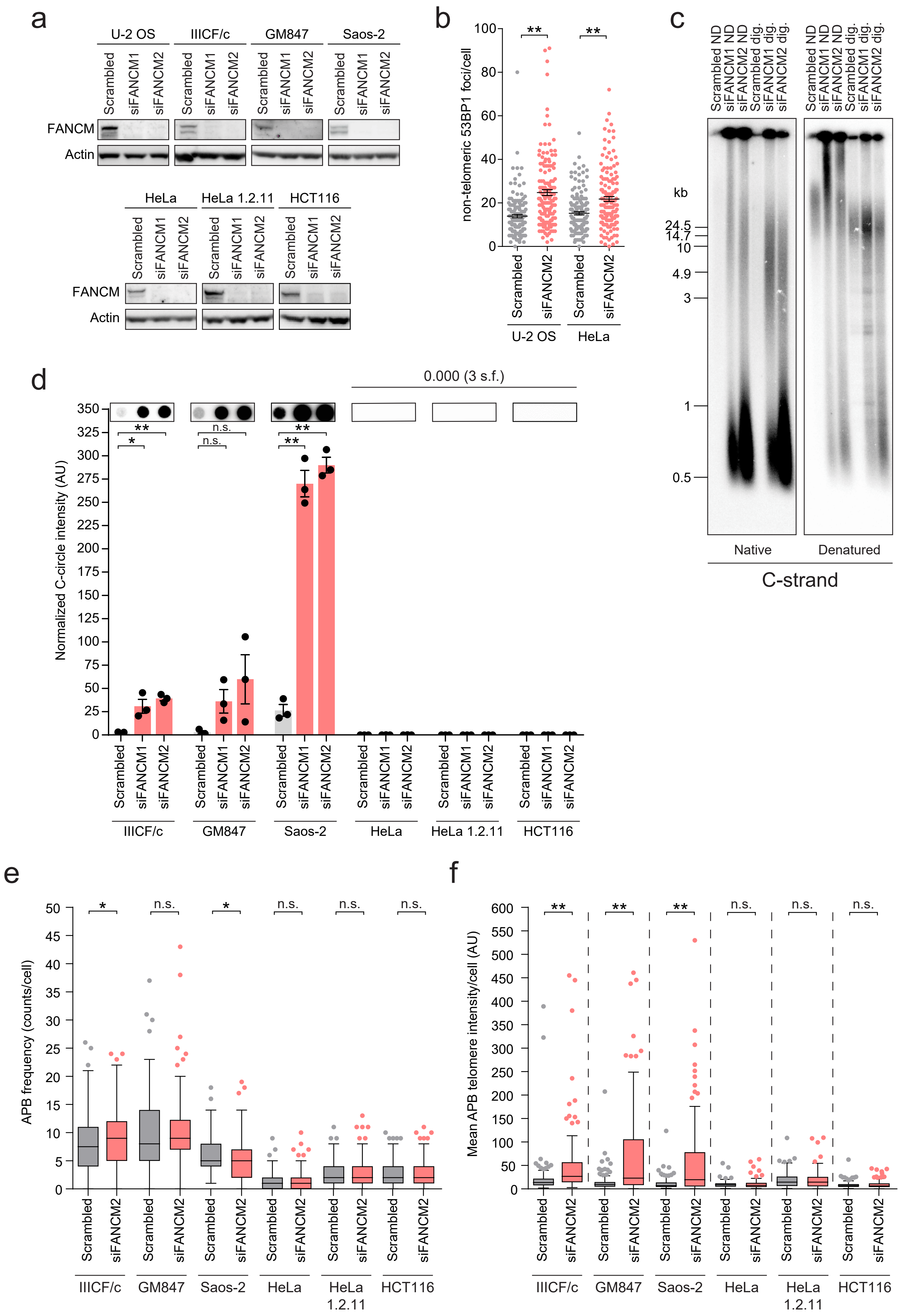

Supplementary Fig. 1. FANCM depletion in telomerase-positive cells. (a) Western immunoblotting of U-2 OS, IIICF/c, GM847, Saos-2, HeLa, HeLa 1.2.11 and HCT116 cells with or without FANCM depletion (siFANCM1 or siFANCM2). **(b)** Quantitation of non-telomeric 53BP1 foci in U-2 OS and HeLa cells with or without FANCM depletion. Scatterplot bars represent the mean ± SEM from n = 150 cells from 3 experiments, $^{**}p < 0.005$, Mann-Whitney test. **(c)** Native and denaturing TRF analysis of non-digested (ND) and HinfI/RsaI digested DNA (dig.) in U-2 OS cells with or without FANCM depletion. **(d)** Representative dot blots and quantitation of C-circles in IIICF/c, GM847, Saos-2, HeLa, HeLa 1.2.11 and HCT116 cells with or without FANCM depletion. C-circles were normalized to a reference sample control. Error bars represent the mean ± SEM from n = 3 experiments, $^{*}p < 0.05$, $^{**}p < 0.005$, Student's t-test. Tukey boxplots of **(e)** APB frequency and **(f)** mean APB telomere foci intensity in IIICF/c, GM847, Saos-2, HeLa, HeLa 1.2.11 and HCT116 cells with or without FANCM depletion. Out of 3 experiments, n = 150 cells scored per treatment, $^{*}p < 0.05$, $^{**}p < 0.005$, n.s. = non-significant, Mann-Whitney test.

Supplementary Fig. 2

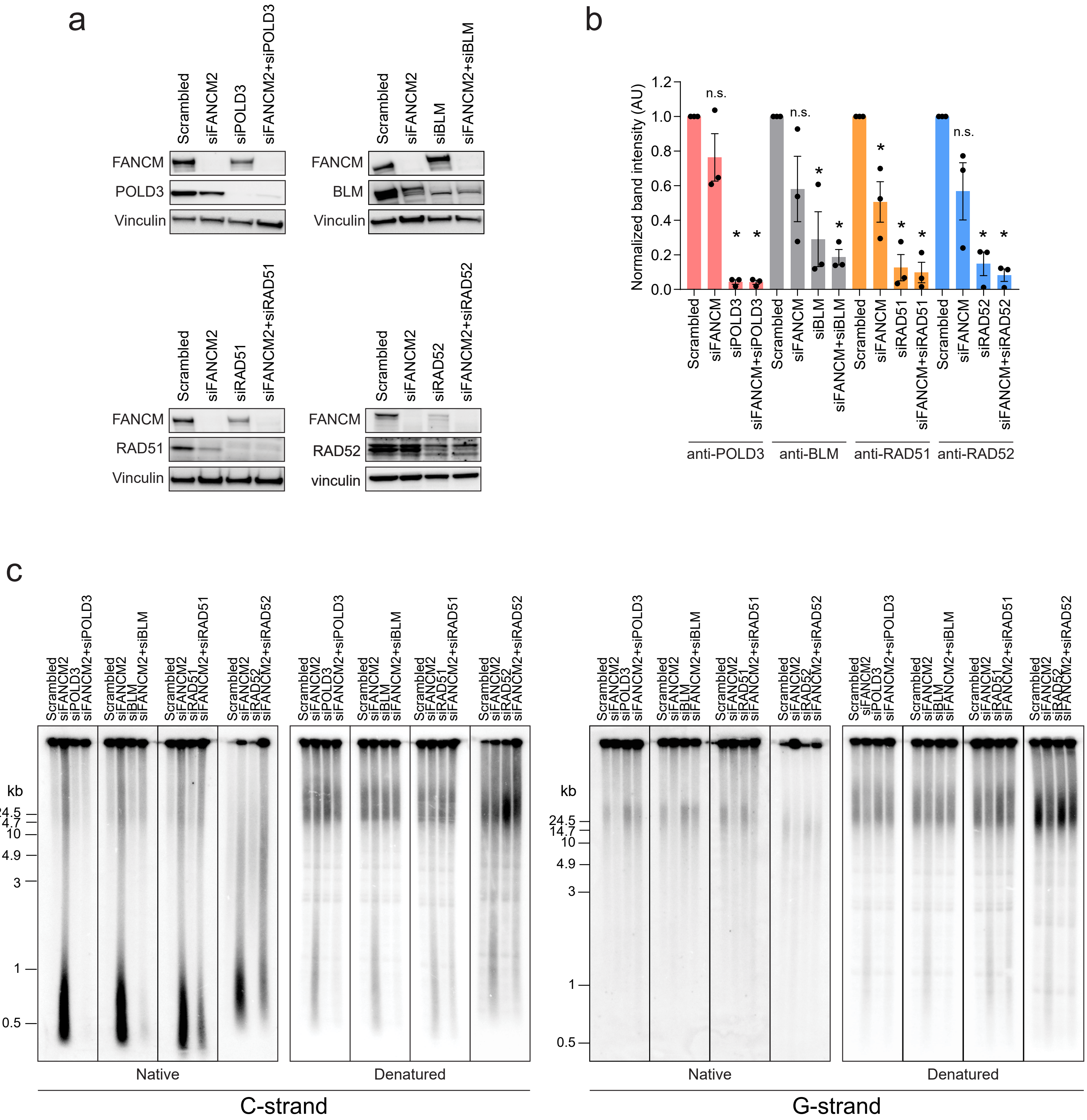

Supplementary Fig. 2. Co-depletion of FANCM and POLD3, BLM, RAD51 or RAD52. (a) Representative Western immunoblotting of U-2 OS cells co-depleted of FANCM (siFANCM2) and either POLD3, BLM, RAD51 or RAD52. **(b)** Densitometry analysis of POLD3, BLM, RAD51 and RAD52 bands from n = 3 experiments. Quantitation was conducted by first normalizing to the loading control (actin or vinculin), then to the scrambled control. Error bars represent the mean ± SEM from n = 3 experiments, *$p < 0.05$, n.s. = non-significant, Student's t-test. **(c)** Native and denaturing TRF analysis of U-2 OS cells co-depleted of FANCM and either POLD3, BLM, RAD51 or RAD52.

Supplementary Fig. 3

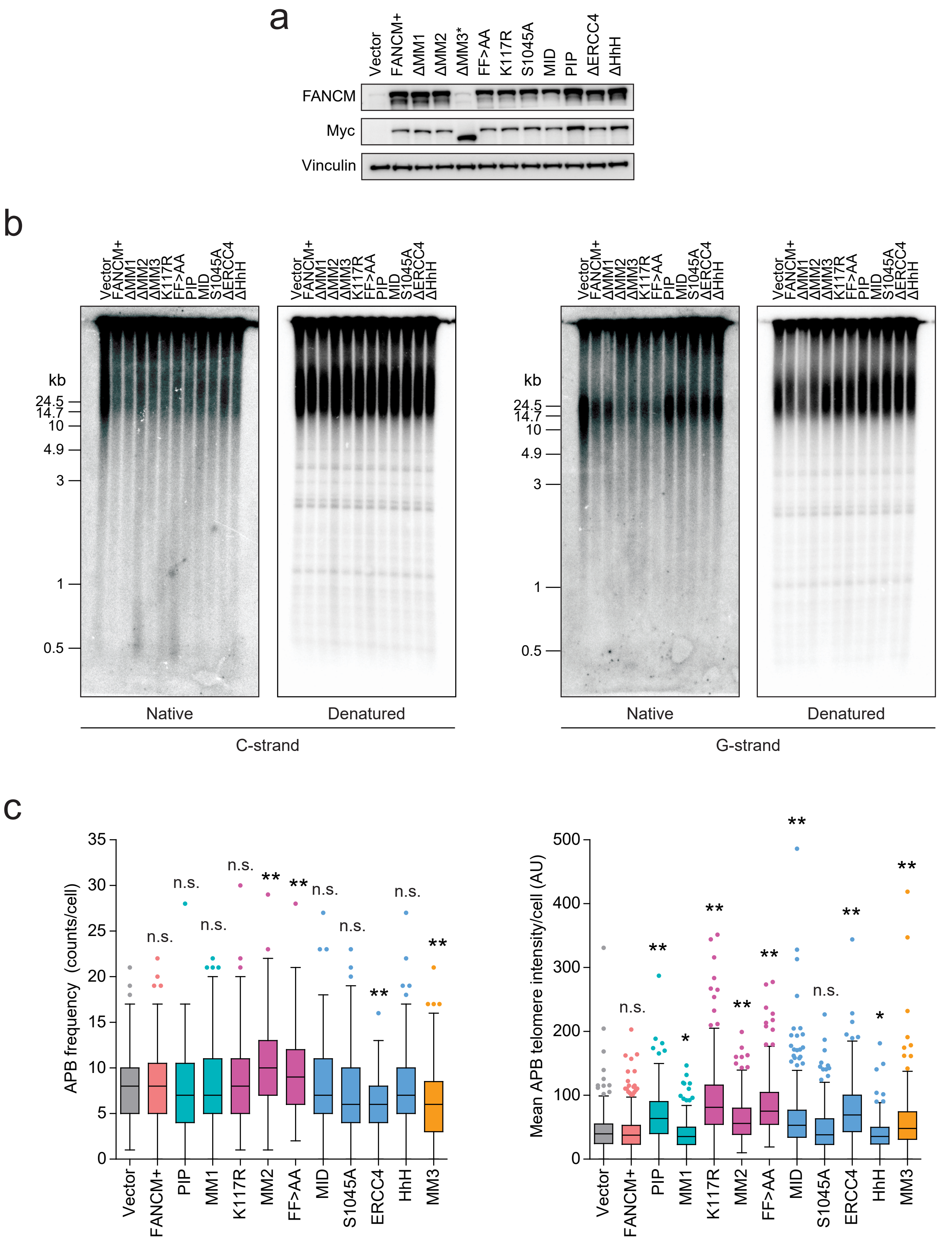

Supplementary Fig. 3. Expression and analysis of FANCM mutants. (a) Western immunoblotting of stable wild-type FANCM (FANCM+) or FANCM mutant overexpression in U-2 OS cells. NB: CV5.1 anti-FANCM mAb binds in the MM3 region and thus does not detect this variant. **(b)** TRF analysis of U-2 OS cells overexpressing wild-type (FANCM+) or FANCM mutants. Gels were hybridized under native and denatured conditions with radiolabelled telomeric probes to detect the C-strand and G-strand. **(c)** Tukey boxplots of APB frequency (left panel) and mean APB telomere intensity (right panel) in U-2 OS cells overexpressing wild-type (FANCM+) or FANCM mutants. Out of 3 experiments, n = 150 cells scored per variant, *$p < 0.05$, **$p < 0.005$, Mann-Whitney test.

Supplementary Fig. 4

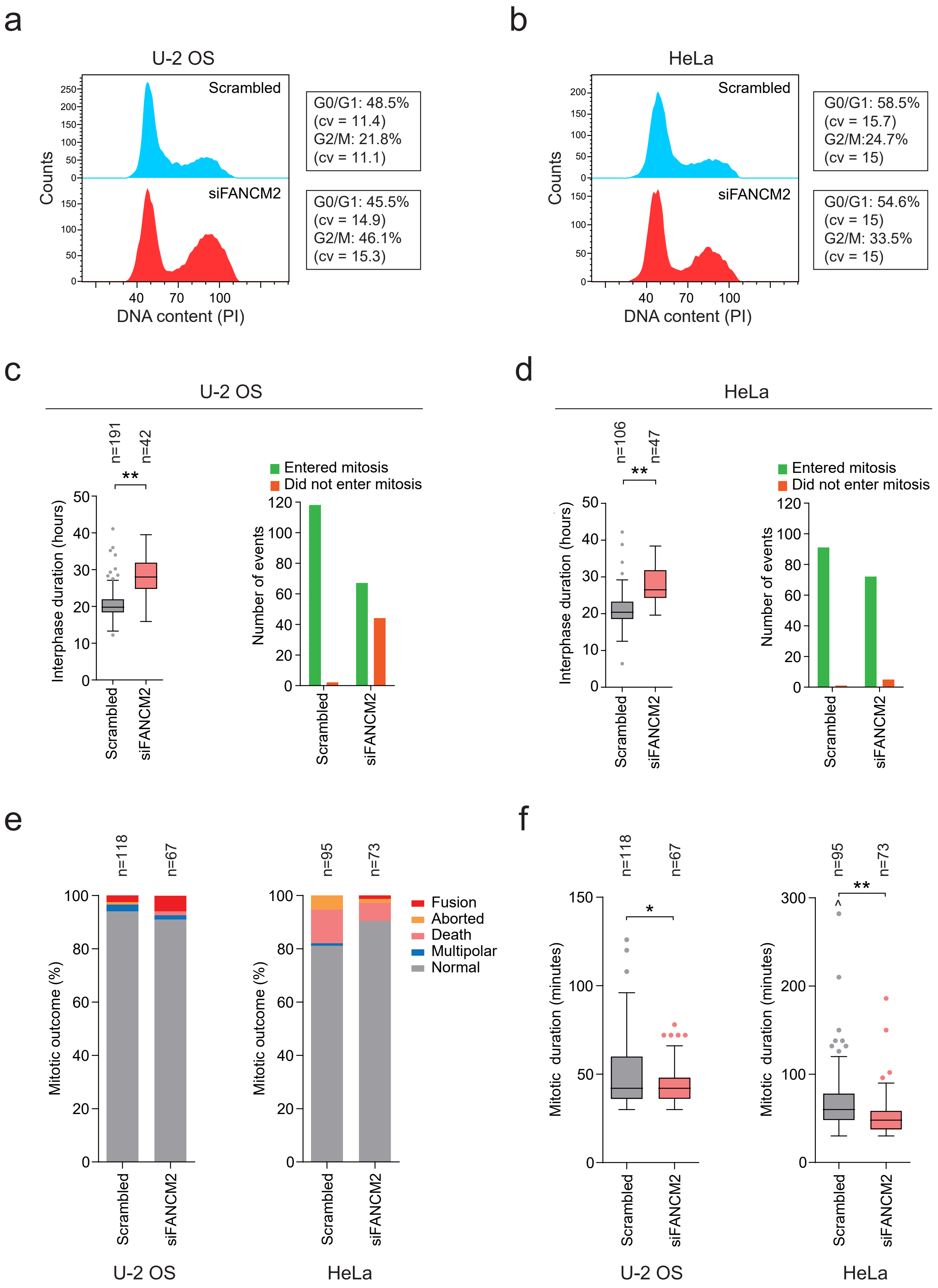

Supplementary Fig. 4. ALT cells are hypersensitive to FANCM depletion. Cell cycle profiles of **(a)** U-2 OS and **(b)** HeLa cells stained with propidium iodide (PI). 9,800-10,000 gated events were collected. Cell cycle distribution was determined by Jett-Dean approximation. Live-cell quantitation of interphase duration (left panel) and mitotic entry (right panel) in **(c)** U-2 OS and **(d)** HeLa cells with or without FANCM depletion. Tukey boxplots represent the quantitation of n = 120 pre-mitotic cells and their daughter cells monitored from 24 h to 72 h post-transfection, $*p < 0.05$, $**p < 0.005$, Mann-Whitney test. Live-cell quantitation of **(e)** mitotic outcomes and **(f)** mitotic duration in U-2 OS and HeLa cells with or without FANCM depletion. Tukey boxplots represent the quantitation of n = 120 pre-mitotic cells from 3 experiments, $*p < 0.05$, $**p < 0.005$, Mann-Whitney test. ^ indicates two data-points outside axis. In c - f, for each dataset, the number of cells analyzed are indicated.

Supplementary Fig. 5

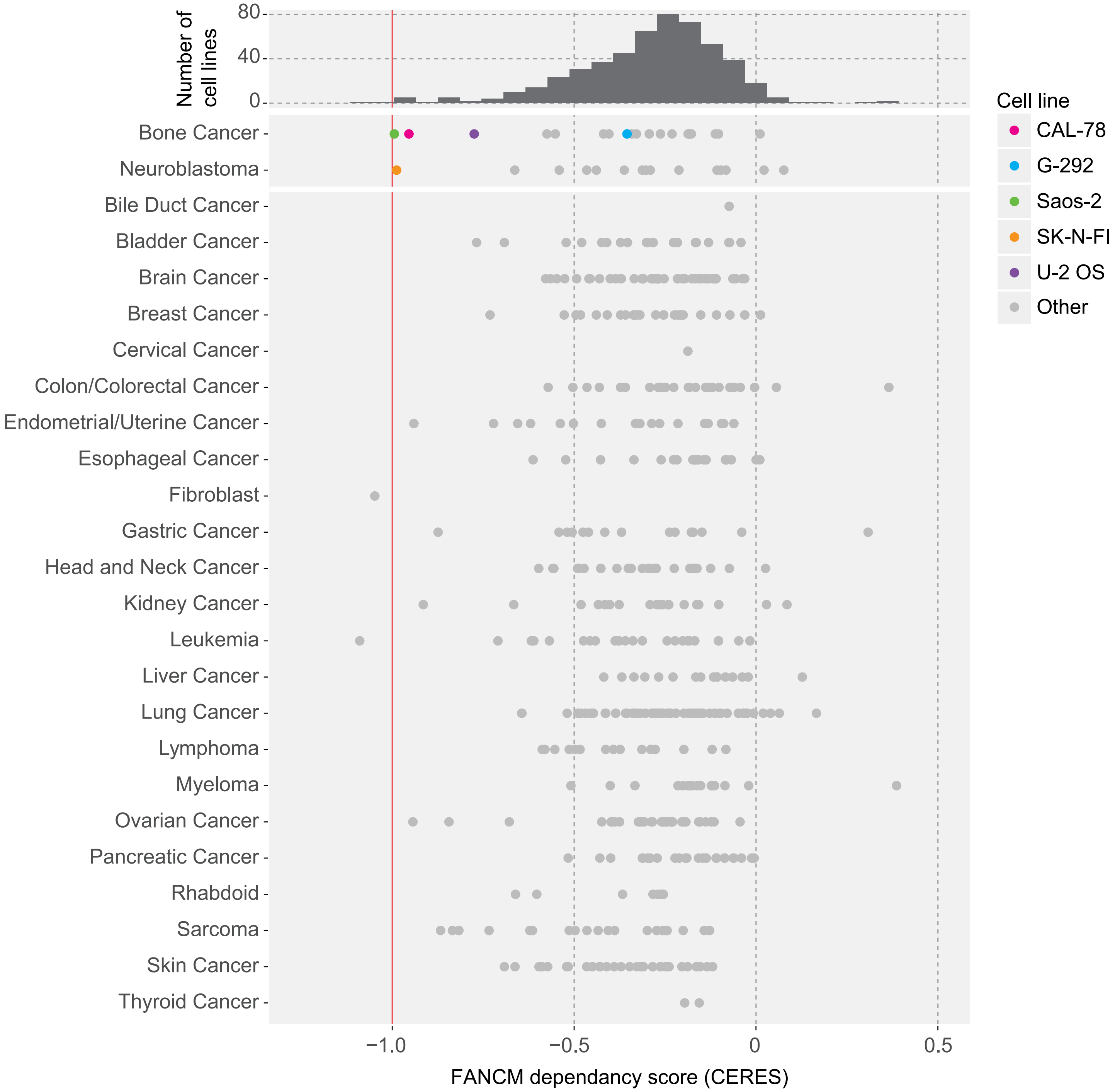

Supplementary Fig. 5. FANCM gene dependency scores across a panel of 517 cancer cell lines from Project Achilles. Gene dependency scores (CERES) for FANCM, determined by CRISPR-Cas9 knockout screens. The dependency score indicates the likelihood that FANCM is essential in the cell line, with a dependency score of 0 indicating that FANCM is not essential for cell viability, and a score of -1 indicating that it is essential. Histogram of FANCM dependency scores across all 517 cancer cell lines (top panel). FANCM dependency scores for each individual cell line, grouped by disease type (bottom panel). Known ALT cell lines have been colored.

Supplementary Fig. 6

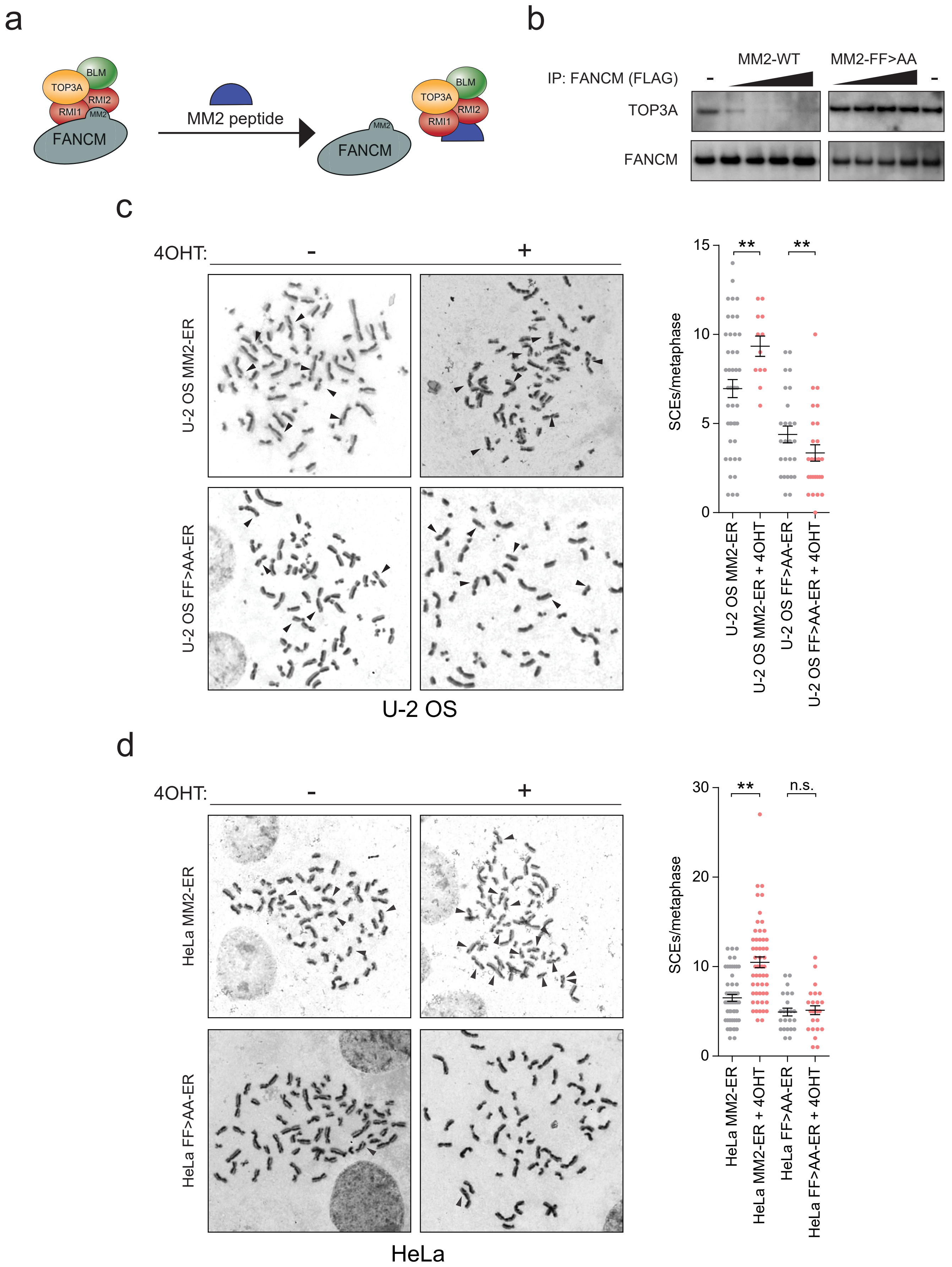

Supplementary Fig. 6. Characterization of the MM2-ER fusion protein. (a) Schematic showing competitive inhibition of FANCM-BTR complex formation by the MM2 peptide. **(b)** Flag-FANCM immunoprecipitation of the FANCM-BTR complex (TOP3A binding) diminishes with increasing concentrations (1-50 μM) of MM2-WT, but not with the MM2-FF>AA mutant peptide in HEK-293 cells expressing Flag-FANCM. **(c)** Representative mitotic spreads showing sister-chromatid exchanges (SCEs) in U-2 OS cells expressing MM2-ER or FF>AA-ER mutant fusion proteins in the presence or absence of 4OHT (left panel). Quantitation of SCEs (right panel). **(d)** Representative mitotic spreads showing sister-chromatid exchanges (SCEs) in HeLa cells expressing MM2-ER or FF>AA-ER mutant fusion proteins in the presence or absence of 4OHT (left panel). Quantitation of SCEs (right panel). SCEs are indicated by black arrows. For c and d, scatterplot bars represent the mean ± SEM number of SCEs from 15-30 mitotic spreads, $^{*}p < 0.05$, $^{**}p < 0.005$, Mann-Whitney test. NB: The frequency of double labelled mitoses in MM2-ER+4OHT samples was approximately 10% of that in the controls, likely due to significant cell cycle delays introduced by activation of the MM2-inhibitor. As thus, SCE levels are likely underestimated in this experiment.

Supplementary Fig. 7

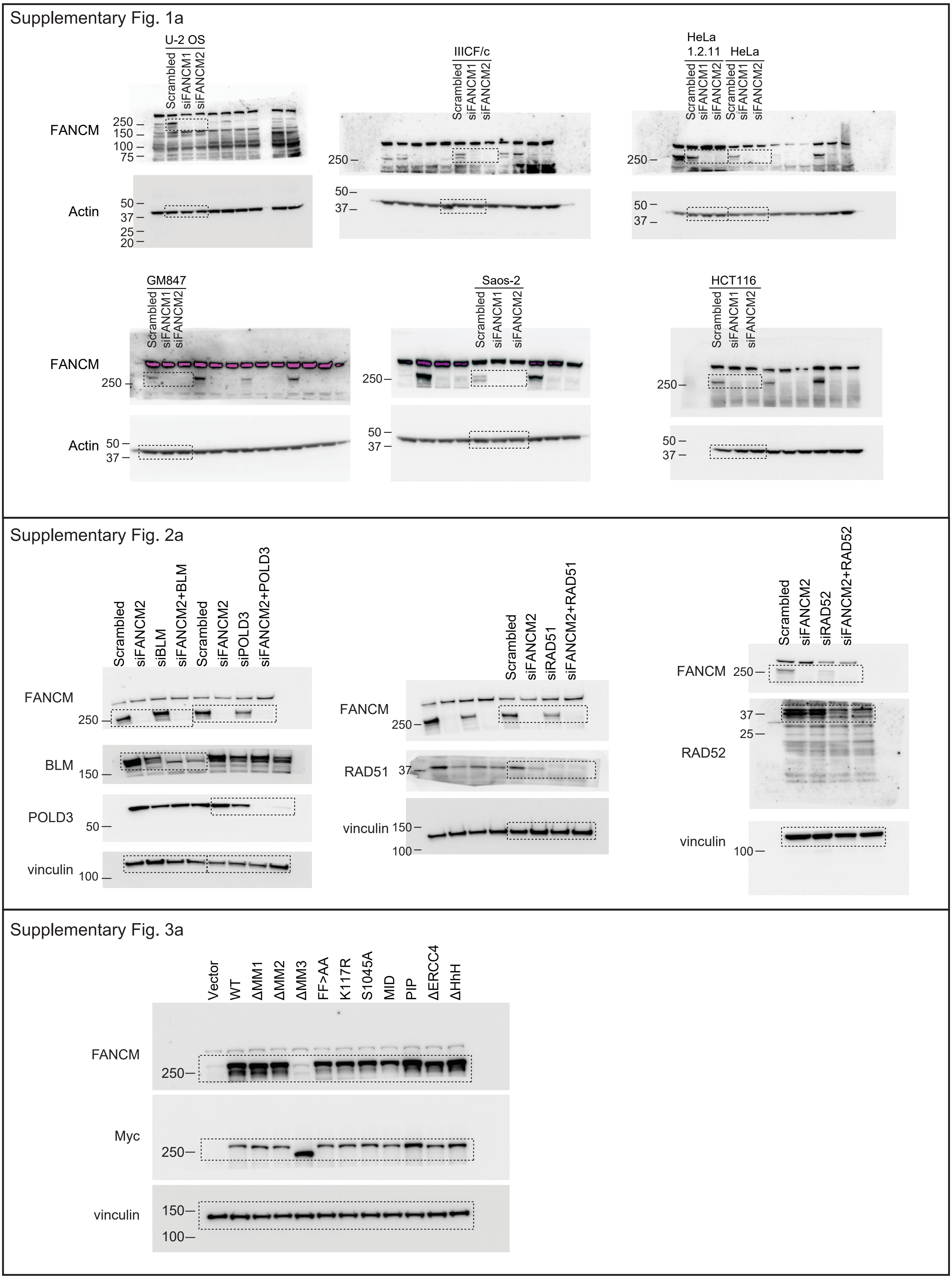

Supplementary Fig. 7. Uncropped Western blots for FANCM depletion and overexpression experiments for corresponding figure panels. Protein size markers have been labelled. Cropped sections of the blots are indicated by dashed-boxes.

Supplementary Fig. 8

Fig. 5b

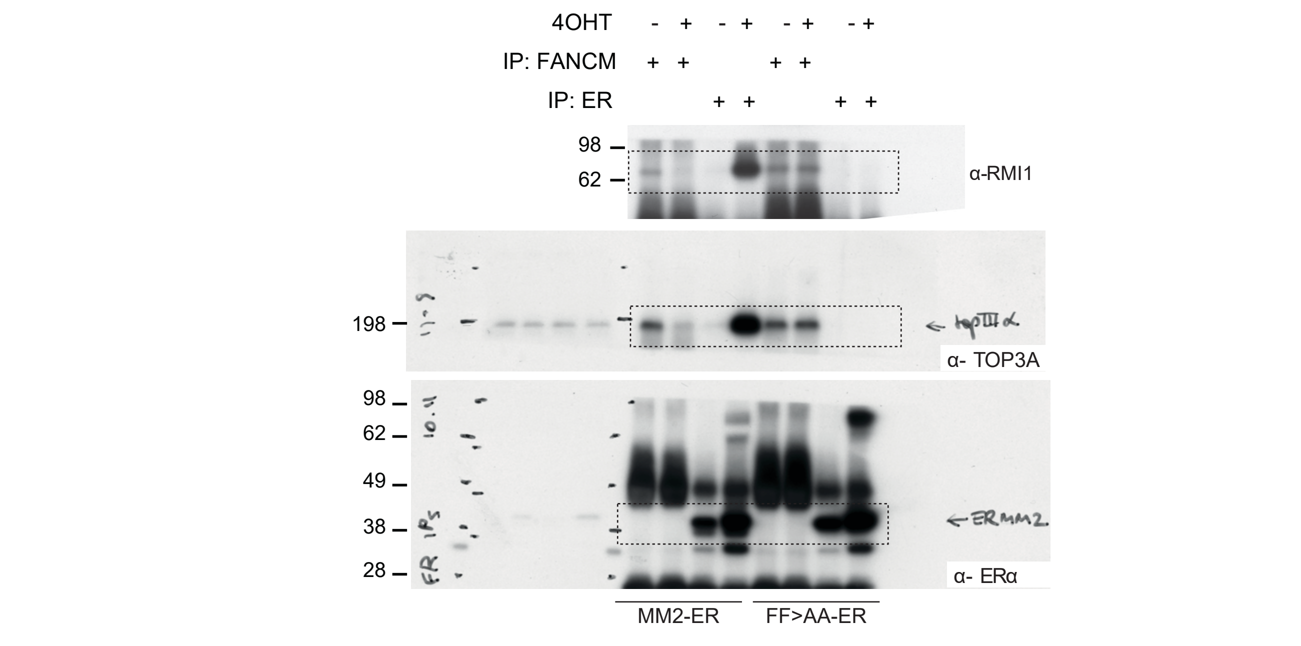

Supplementary Fig. 6b

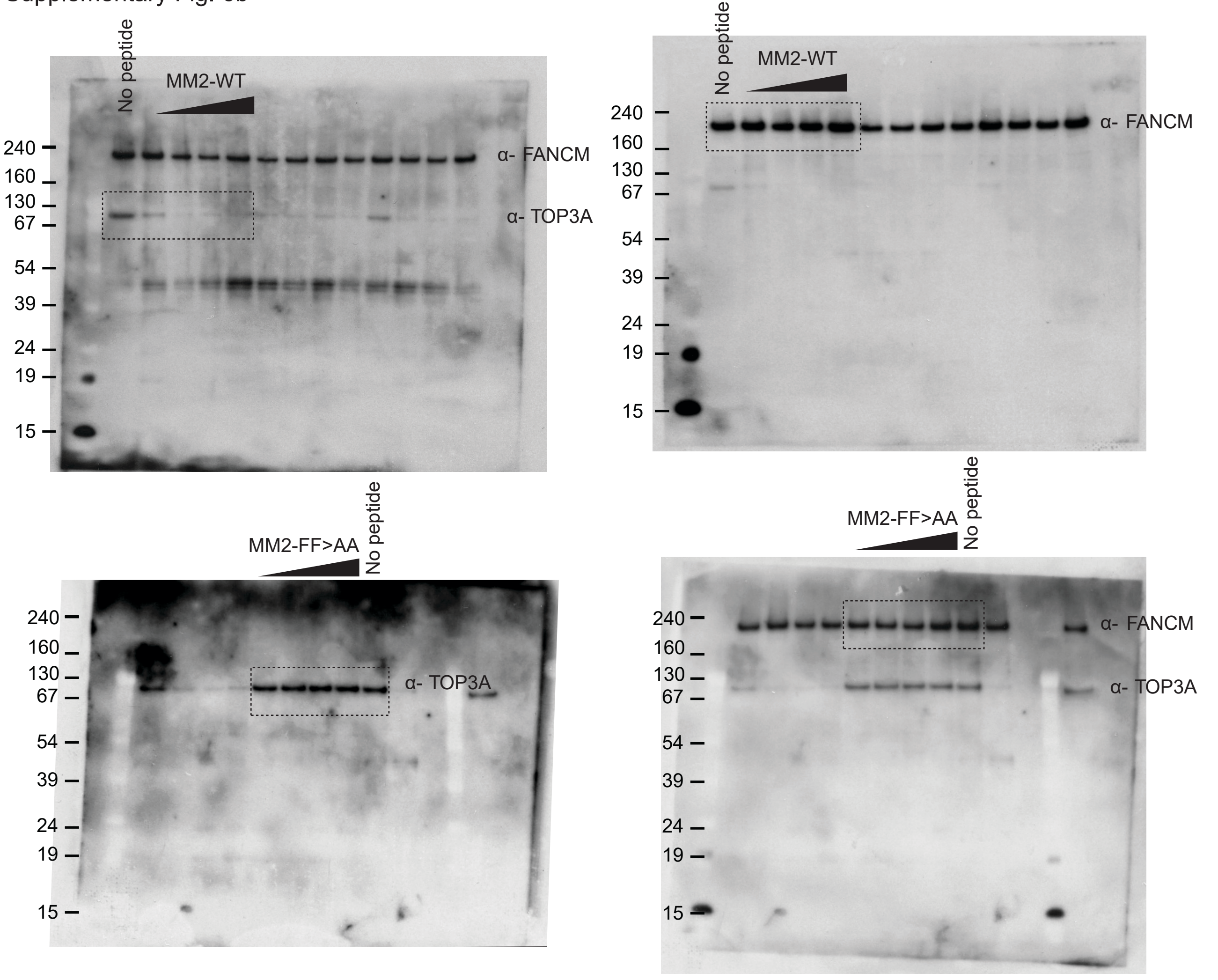

Supplementary Fig. 8. Uncropped Western blots for MM2-ER, FF>AA-ER fusion peptide experiments for corresponding figure panels. Protein size markers have been labelled. Cropped sections of the blots are indicated by dashed-boxes.

Supplementary Fig. 9

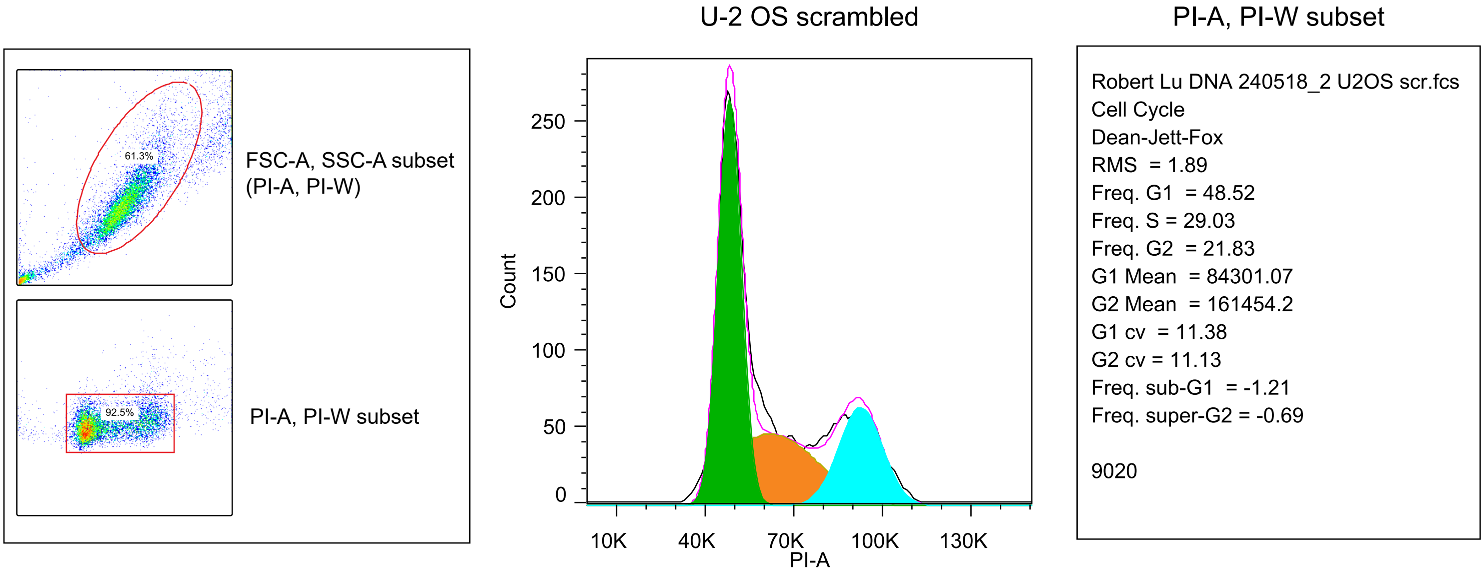

Supplementary Fig. 9. Example of gating strategy performed for propidium-iodide cell cycle analysis. Pseudo-colored plot of SSC-A, FSC-A subset gating followed by PI-A, PI-W subset gating (left panel). PI-A, PI-W subset was then subject to Dean-Jett cell cycle analysis. Gates for G0/G1, S and G2/M phases are indicated in green, orange and cyan, respectively (middle panel) with associated statistics (right panel) and the curve of best fit indicated in violet (middle panel).

Supplementary Table 1: List of antibodies used in this study

Target	Species	Source, Cat #	Western Blot Dilution	Immunofluorescence Dilution	Immunoprecipitation Amount
**Actin**	Rabbit	Sigma Aldrich, A2066	1:5000	-	-
**Vinculin**	Mouse	Sigma Aldrich, V9131	1 µg/ml	-	-
**FANCM**	Mouse	CV5.1 (Vuono et al., 2016) Novus, NBP2-50418	1:1000	-	-
**FANCM**	Rabbit	Abcam, ab35620	-	-	1 µg
**TRF2**	Rabbit	Novus, NB110-57130	-	1:200	-
**Myc-tag**	Mouse	Cell Signalling Technologies, 2276	1:1000	-	-
**FLAG (DDK)**	Mouse	Aviva Systems Biology, OAEA00002	-	-	400 ng
**PML**	Goat	Santa Cruz	-	1:400	-
**γ-H2AX**	Mouse	Merck Millipore	-	1:500	-
**BLM**	Rabbit	Bethyl, A300-110A	1:1000	-	-
**POLD3**	Mouse	Novus, H00010714-M01	1:1000	1:200	-
**RAD51**	Mouse	Abcam, ab213	1:1000	-	-
**RAD52**	Mouse	Santa Cruz, SC-365341	1:1000	-	-

**RMI1**	Mouse	Abnova, H00080010-B02P	1:1000	-	-
**ERα (MC-20)**	Rabbit	Santa Cruz, sc-542	1:1000	-	400 ng
**TOP3A**	Rabbit	D6 (Wu et al., 2000)	1:1000	-	-
**Anti-FLAG M2 Peroxidase (HRP)**	Mouse	Sigma Aldrich, A8592	1:2500	-	-
**Anti-BrdU (cross-reacts with CldU)**	Rat	Bio Rad, OBT0030	-	1:25	-
**Anti-BrdU**	Mouse	BD Biosciences, 347580	-	-	2 µg
