# Supplementary material for: The FANCM-BLM-TOP3A-RMI complex suppresses alternative lengthening of telomeres (ALT)
Source: Nat Commun. 2019 May 28;10:2252. doi: 10.1038/s41467-019-10180-6 (PMC6538672; doi:10.1038/s41467-019-10180-6)
Supplement: Supplementary file 3 — Reporting Summary [file 41467_2019_10180_MOESM3_ESM.pdf]

nature research

Corresponding author(s): Hilda A. Pickett

Reporting Summary

Nature Research wishes to improve the reproducibility of the work that we publish. This form provides structure for consistency and transparency in reporting. For further information on Nature Research policies, see Authors & Referees and the Editorial Policy Checklist.

Statistical parameters

When statistical analyses are reported, confirm that the following items are present in the relevant location (e.g. figure legend, table legend, main text, or Methods section).

n/a	Confirmed	
☐	☒	The exact sample size (n) for each experimental group/condition, given as a discrete number and unit of measurement
☐	☒	An indication of whether measurements were taken from distinct samples or whether the same sample was measured repeatedly
☐	☒	The statistical test(s) used AND whether they are one- or two-sided<br>*Only common tests should be described solely by name; describe more complex techniques in the Methods section.*
☐	☒	A description of all covariates tested
☒	☐	A description of any assumptions or corrections, such as tests of normality and adjustment for multiple comparisons
☐	☒	A full description of the statistics including central tendency (e.g. means) or other basic estimates (e.g. regression coefficient) AND variation (e.g. standard deviation) or associated estimates of uncertainty (e.g. confidence intervals)
☐	☒	For null hypothesis testing, the test statistic (e.g. F, t, r) with confidence intervals, effect sizes, degrees of freedom and P value noted<br>*Give P values as exact values whenever suitable.*
☒	☐	For Bayesian analysis, information on the choice of priors and Markov chain Monte Carlo settings
☒	☐	For hierarchical and complex designs, identification of the appropriate level for tests and full reporting of outcomes
☒	☐	Estimates of effect sizes (e.g. Cohen's d, Pearson's r), indicating how they were calculated
☐	☒	Clearly defined error bars<br>*State explicitly what error bars represent (e.g. SD, SE, CI)*

Our web collection on statistics for biologists may be useful.

Software and code

Policy information about availability of computer code

Data collection	Microsoft Excel
Data analysis	Cellprofiler v2.1.1

For manuscripts utilizing custom algorithms or software that are central to the research but not yet described in published literature, software must be made available to editors/reviewers upon request. We strongly encourage code deposition in a community repository (e.g. GitHub). See the Nature Research guidelines for submitting code & software for further information.

Data

Policy information about availability of data

All manuscripts must include a data availability statement. This statement should provide the following information, where applicable:

- Accession codes, unique identifiers, or web links for publicly available datasets
- A list of figures that have associated raw data
- A description of any restrictions on data availability

The data that support the findings of this study are available from the corresponding author upon reasonable request.

Field-specific reporting

Please select the best fit for your research. If you are not sure, read the appropriate sections before making your selection.

☒ Life sciences ☐ Behavioural & social sciences ☐ Ecological, evolutionary & environmental sciences

For a reference copy of the document with all sections, see nature.com/authors/policies/ReportingSummary-flat.pdf

Life sciences study design

All studies must disclose on these points even when the disclosure is negative.

Sample size	Sample sizes were chosen based on previous publications in the field.
Data exclusions	No data were excluded from the analysis.
Replication	All experiments were conducted with n=3 biological replicates.
Randomization	Experiments were performed in non-clonal cell lines.
Blinding	Automated image analysis was used where appropriate to minimize bias.

Reporting for specific materials, systems and methods

Materials & experimental systems

n/a	Involved in the study	
☐	☒	Unique biological materials
☐	☒	Antibodies
☐	☒	Eukaryotic cell lines
☒	☐	Palaeontology
☒	☐	Animals and other organisms
☒	☐	Human research participants

Methods

n/a	Involved in the study	
☒	☐	ChIP-seq
☐	☒	Flow cytometry
☒	☐	MRI-based neuroimaging

Unique biological materials

Policy information about availability of materials

Obtaining unique materials	Unique biological materials will be provided upon reasonable request.

Antibodies

Antibodies used	Information provided in Supplementary Table 1.
Validation	Uncropped Western blots provided in Supplementary Information and Source Data. Additional information provided on the manufacturer's website.

Eukaryotic cell lines

Policy information about cell lines

Cell line source(s)	U-2 OS, Saos-2 and HeLa were obtained from the American Type Culture Collection. HEK293 and HCT116 were obtained from the Francis Crick Institute. IIICF/c is a spontaneously immortalized breast fibroblast line from an individual with Li-Fraumeni syndrome. HeLa1.2.11 were obtained from Dr Tony Cesare. GM847 were obtained from Olivia Pereira-Smith.
Authentication	Cell lines were authenticated by 16-locus short-tandem-repeat profiling by CellBank Australia (Children's Medical Research Institute).
Mycoplasma contamination	Cell lines were tested for mycoplasma contamination by CellBank Australia (Children's Medical Research Institute).

Commonly misidentified lines (See ICLAC register) | No commonly misidentified cell lines were used.

Flow Cytometry

Plots

Confirm that:

☒ The axis labels state the marker and fluorochrome used (e.g. CD4-FITC).

☒ The axis scales are clearly visible. Include numbers along axes only for bottom left plot of group (a 'group' is an analysis of identical markers).

☒ All plots are contour plots with outliers or pseudocolor plots.

☒ A numerical value for number of cells or percentage (with statistics) is provided.

Methodology

Sample preparation	ethanol fixation followed by propidium iodide staining
Instrument	BD FaCSCanto I
Software	BD FACSDiva, FlowJo v7.5
Cell population abundance	9,800 - 10,000 P1 events
Gating strategy	P1: Gate for cell cluster on SSC-A, FSC-A bivariate plot. P2 (final population): Gate out doublets on PI-W, PI-A bivariate plot.

☒ Tick this box to confirm that a figure exemplifying the gating strategy is provided in the Supplementary Information.
