# Supplementary material for: The FANCM-BLM-TOP3A-RMI complex suppresses alternative lengthening of telomeres (ALT)
Source: Nat Commun. 2019 May 28;10:2252. doi: 10.1038/s41467-019-10180-6 (PMC6538672; doi:10.1038/s41467-019-10180-6)
Supplement: Supplementary file 4 — Source Data [file 41467_2019_10180_MOESM4_ESM.zip › Supp Fig 2c.pptx]

## Slide 1
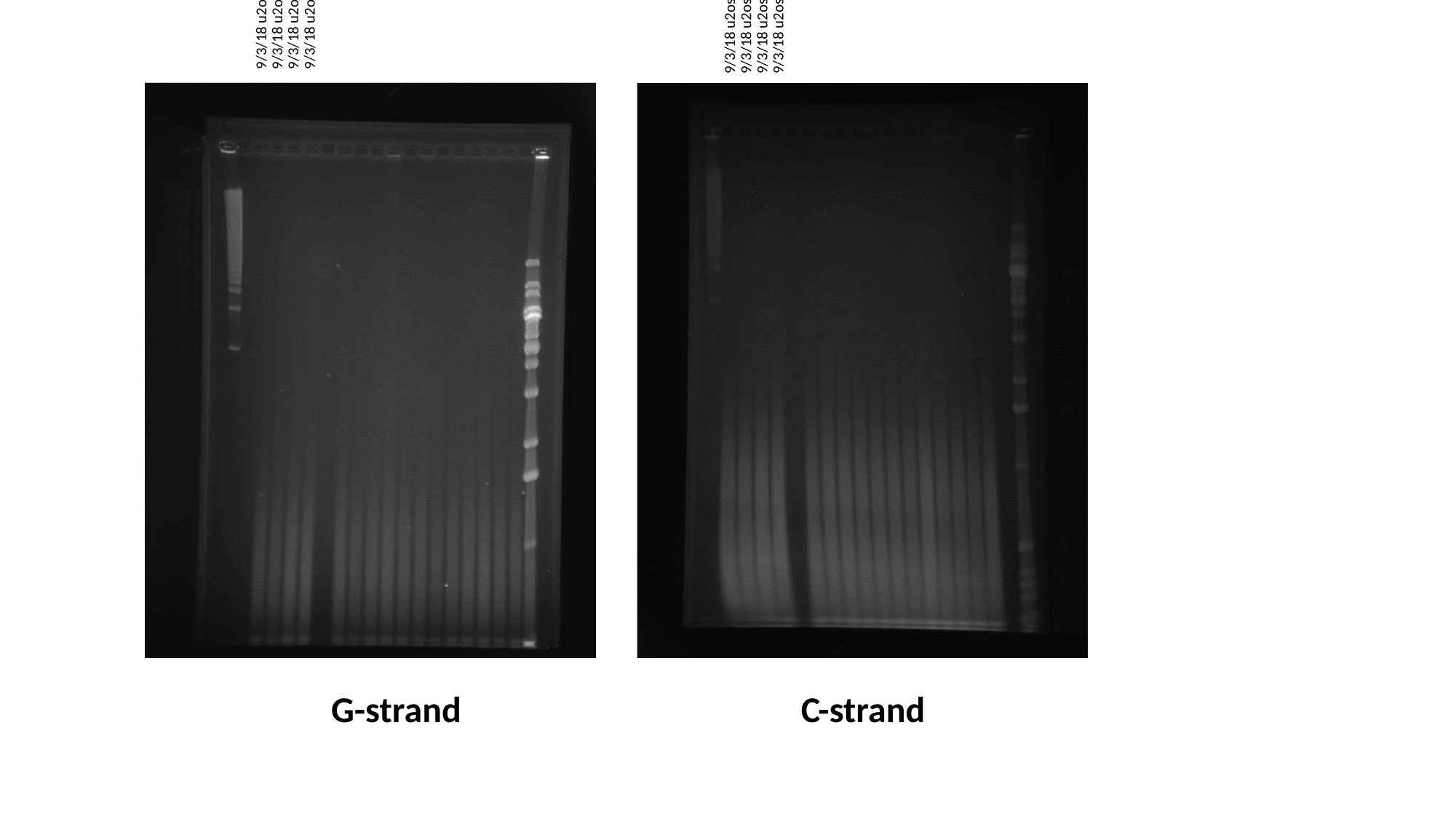

| 9/3/18 u2os si-scr | 9/3/18 u2os si-fancm | 9/3/18 u2os si-rad52 | 9/3/18 u2os si-fancm+rad52 |
| --- | --- | --- | --- |
| 9/3/18 u2os si-scr | 9/3/18 u2os si-fancm | 9/3/18 u2os si-rad52 | 9/3/18 u2os si-fancm+rad52 |
| --- | --- | --- | --- |
C-strand
G-strand

## Slide 2
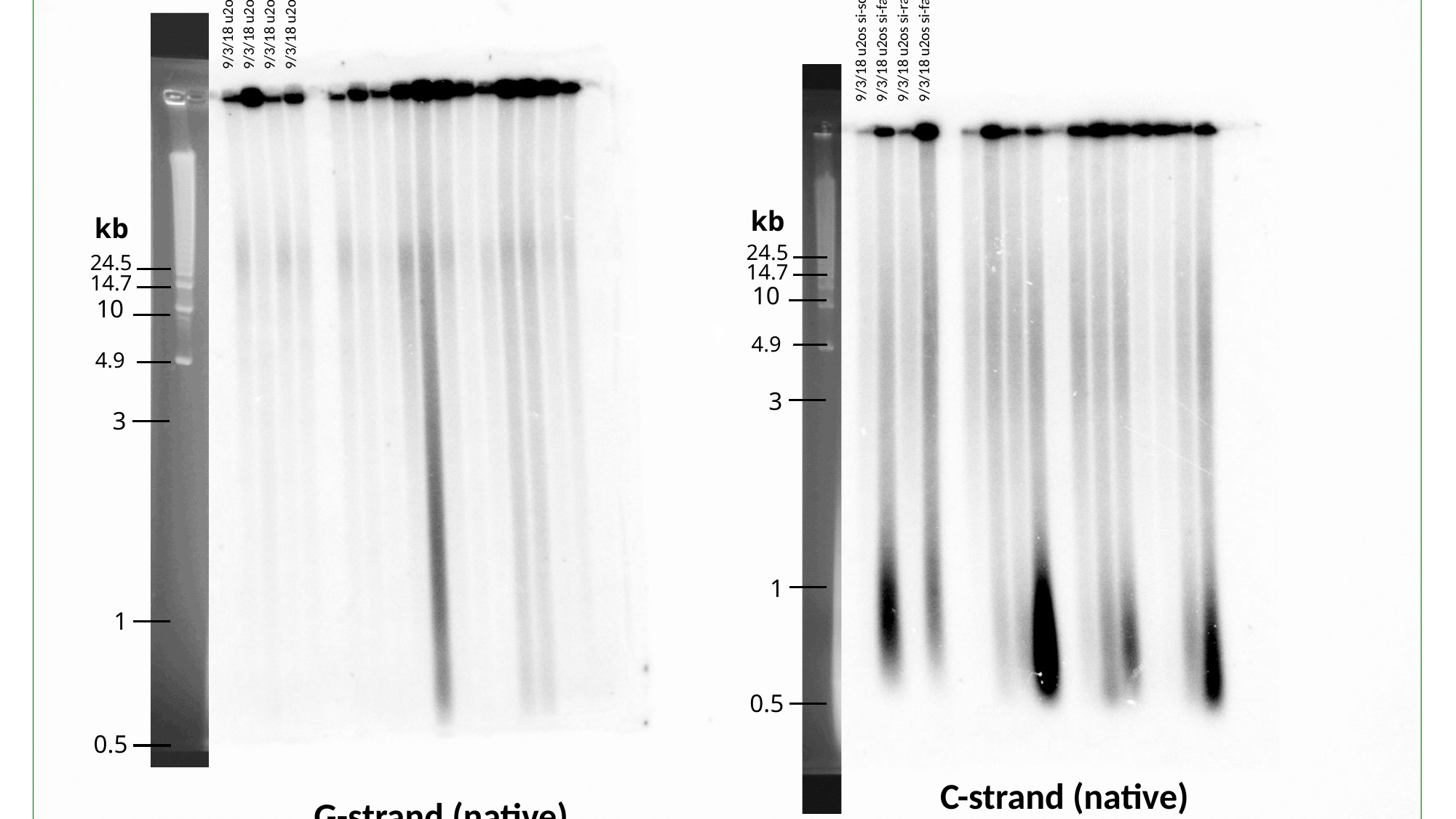

| 9/3/18 u2os si-scr | 9/3/18 u2os si-fancm | 9/3/18 u2os si-rad52 | 9/3/18 u2os si-fancm+rad52 |
| --- | --- | --- | --- |
| 9/3/18 u2os si-scr | 9/3/18 u2os si-fancm | 9/3/18 u2os si-rad52 | 9/3/18 u2os si-fancm+rad52 |
| --- | --- | --- | --- |
kb
24.5
14.7
4.9
10
3
1
0.5
kb
24.5
14.7
4.9
10
3
1
0.5
C-strand (native)
G-strand (native)

## Slide 3
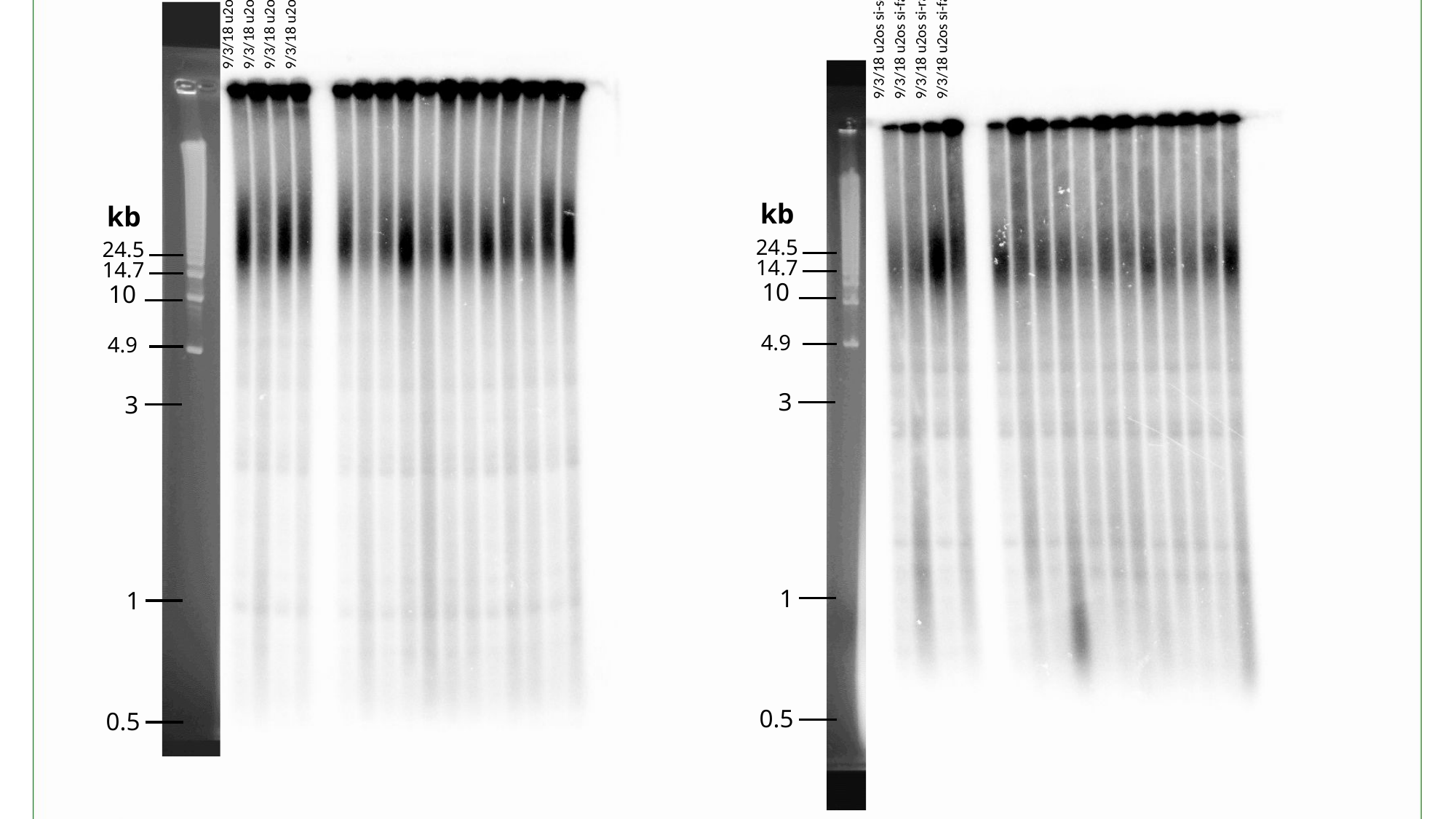

| 9/3/18 u2os si-scr | 9/3/18 u2os si-fancm | 9/3/18 u2os si-rad52 | 9/3/18 u2os si-fancm+rad52 |
| --- | --- | --- | --- |
| 9/3/18 u2os si-scr | 9/3/18 u2os si-fancm | 9/3/18 u2os si-rad52 | 9/3/18 u2os si-fancm+rad52 |
| --- | --- | --- | --- |
kb
24.5
14.7
4.9
10
3
1
0.5
kb
24.5
14.7
4.9
10
3
1
0.5
C-strand (denatured)
G-strand (denatured)

## Slide 4
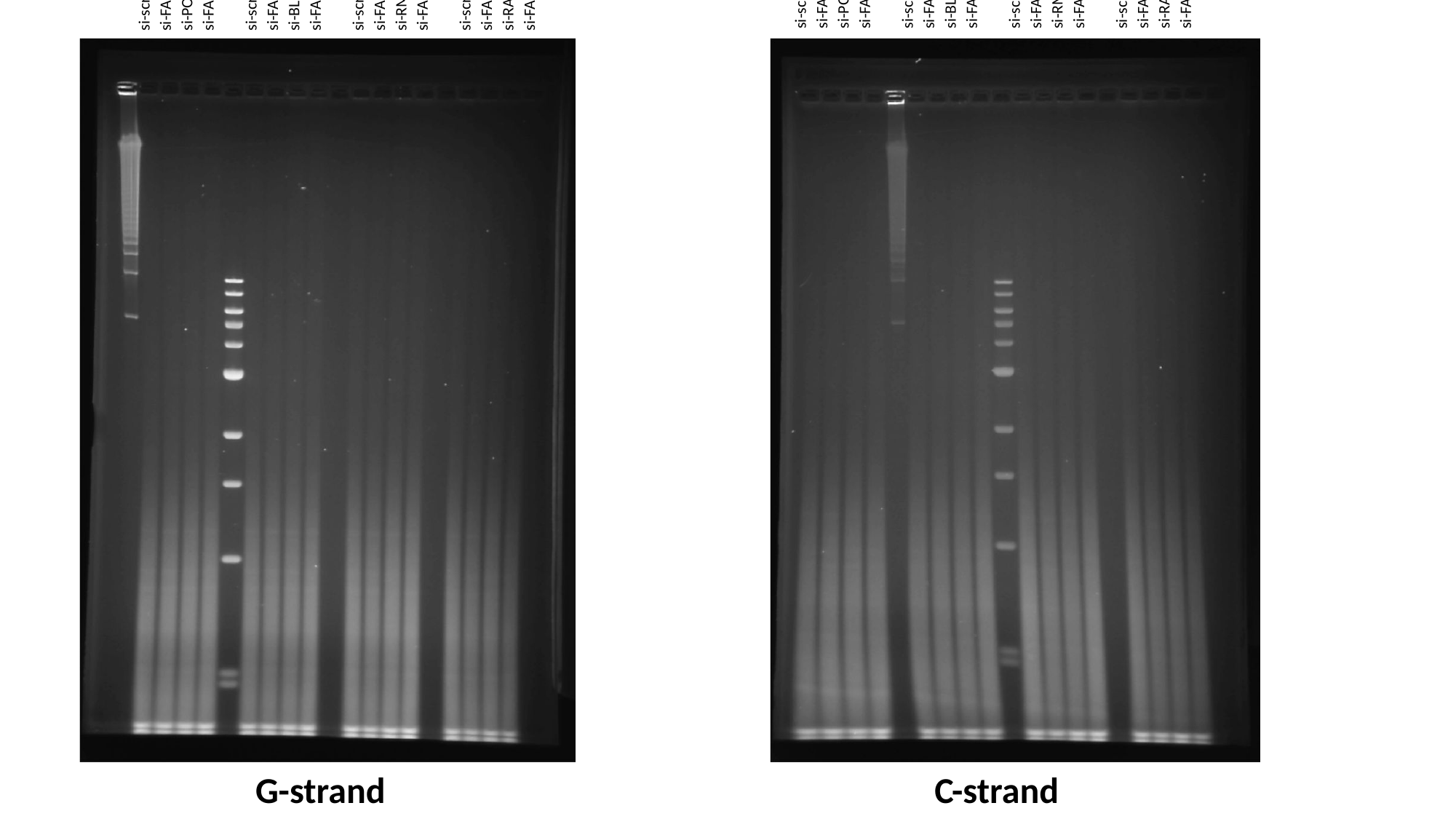

| si-scr | si-FANCM | si-POLD3 | si-FANCM+POLD3 | | si-scr | si-FANCM | si-BLM | si-FANCM+BLM | | si-scr | si-FANCM | si-RMI1 | si-FANCM+RMI1 | | si-scr | si-FANCM | si-RAD51 | si-FANCM+RAD51 |
| --- | --- | --- | --- | --- | --- | --- | --- | --- | --- | --- | --- | --- | --- | --- | --- | --- | --- | --- |
| si-scr | si-FANCM | si-POLD3 | si-FANCM+POLD3 | | si-scr | si-FANCM | si-BLM | si-FANCM+BLM | | si-scr | si-FANCM | si-RMI1 | si-FANCM+RMI1 | | si-scr | si-FANCM | si-RAD51 | si-FANCM+RAD51 |
| --- | --- | --- | --- | --- | --- | --- | --- | --- | --- | --- | --- | --- | --- | --- | --- | --- | --- | --- |
C-strand
G-strand

## Slide 5
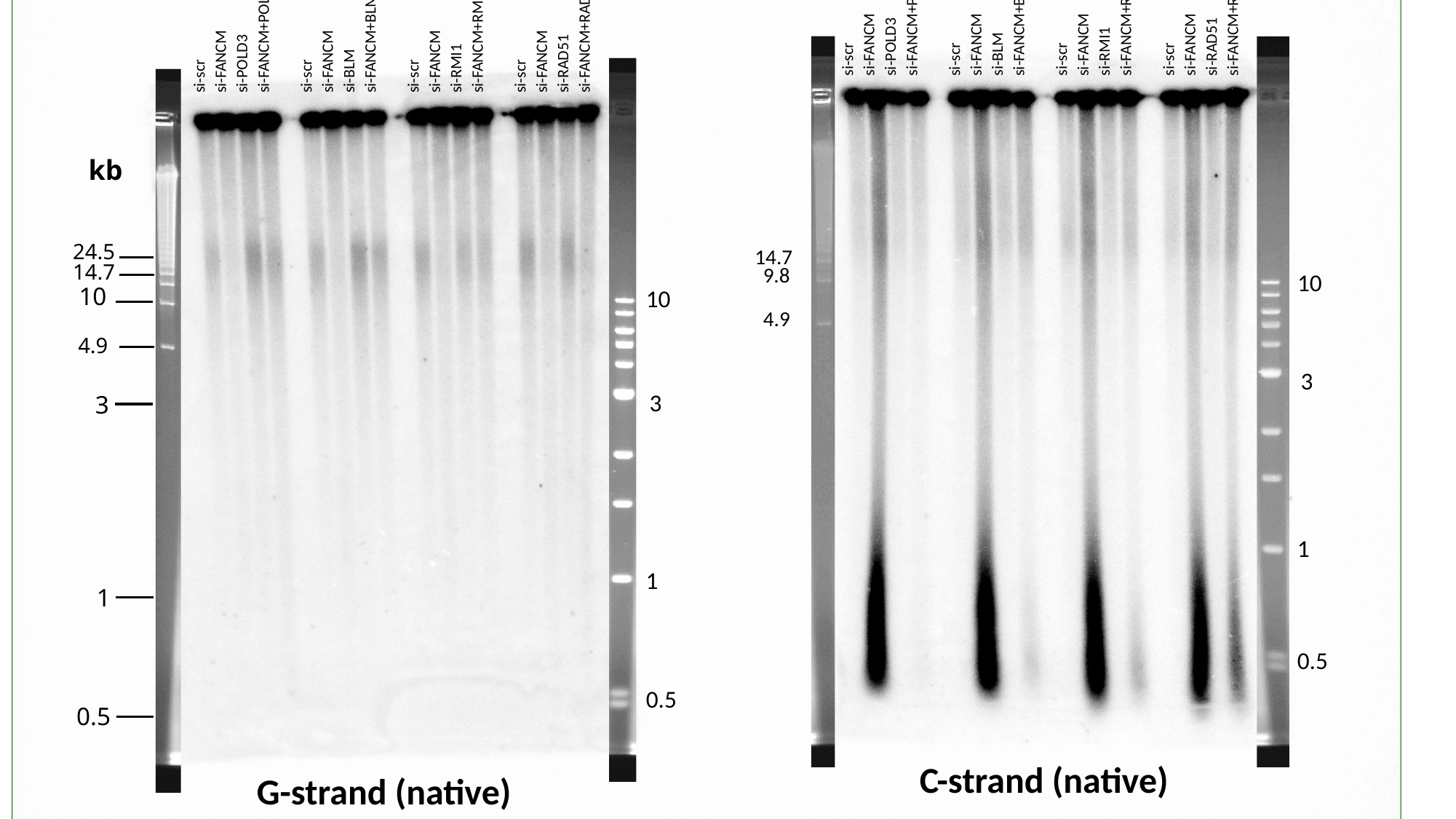

| si-scr | si-FANCM | si-POLD3 | si-FANCM+POLD3 | | si-scr | si-FANCM | si-BLM | si-FANCM+BLM | | si-scr | si-FANCM | si-RMI1 | si-FANCM+RMI1 | | si-scr | si-FANCM | si-RAD51 | si-FANCM+RAD51 |
| --- | --- | --- | --- | --- | --- | --- | --- | --- | --- | --- | --- | --- | --- | --- | --- | --- | --- | --- |
| si-scr | si-FANCM | si-POLD3 | si-FANCM+POLD3 | | si-scr | si-FANCM | si-BLM | si-FANCM+BLM | | si-scr | si-FANCM | si-RMI1 | si-FANCM+RMI1 | | si-scr | si-FANCM | si-RAD51 | si-FANCM+RAD51 |
| --- | --- | --- | --- | --- | --- | --- | --- | --- | --- | --- | --- | --- | --- | --- | --- | --- | --- | --- |
kb
24.5
14.7
10
4.9
3
1
0.5
14.7
9.8
4.9
10
3
1
0.5
10
3
1
0.5
C-strand (native)
G-strand (native)

## Slide 6
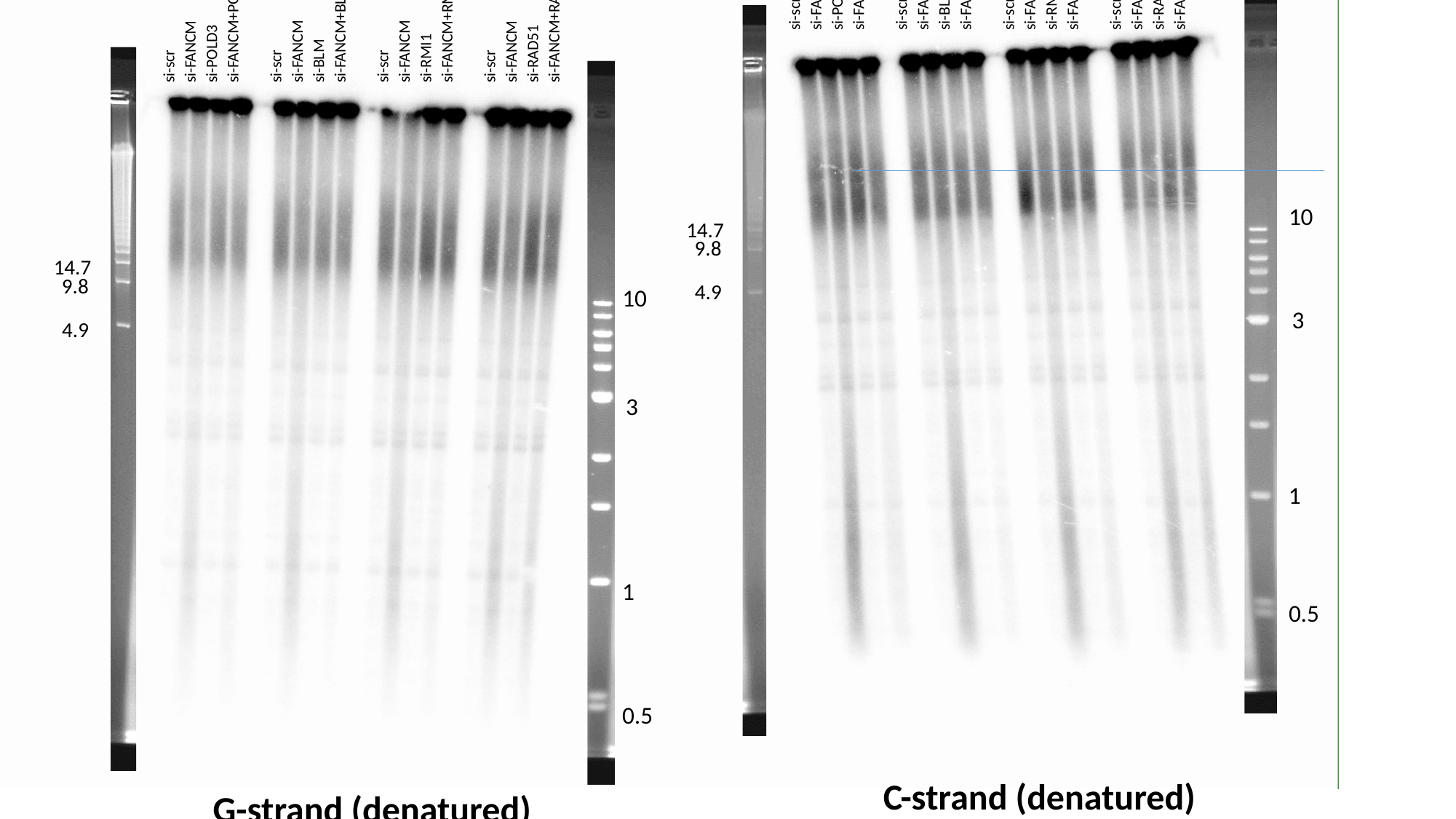

| si-scr | si-FANCM | si-POLD3 | si-FANCM+POLD3 | | si-scr | si-FANCM | si-BLM | si-FANCM+BLM | | si-scr | si-FANCM | si-RMI1 | si-FANCM+RMI1 | | si-scr | si-FANCM | si-RAD51 | si-FANCM+RAD51 |
| --- | --- | --- | --- | --- | --- | --- | --- | --- | --- | --- | --- | --- | --- | --- | --- | --- | --- | --- |
| si-scr | si-FANCM | si-POLD3 | si-FANCM+POLD3 | | si-scr | si-FANCM | si-BLM | si-FANCM+BLM | | si-scr | si-FANCM | si-RMI1 | si-FANCM+RMI1 | | si-scr | si-FANCM | si-RAD51 | si-FANCM+RAD51 |
| --- | --- | --- | --- | --- | --- | --- | --- | --- | --- | --- | --- | --- | --- | --- | --- | --- | --- | --- |
10
3
1
0.5
14.7
9.8
4.9
14.7
9.8
4.9
10
3
1
0.5
C-strand (denatured)
G-strand (denatured)
